# Repression of enhancer RNA PHLDA1 promotes tumorigenesis and progression of Ewing sarcoma *via* decreasing infiltrating T‐lymphocytes: A bioinformatic analysis

**DOI:** 10.3389/fgene.2022.952162

**Published:** 2022-08-25

**Authors:** Runzhi Huang, Dan Huang, Siqiao Wang, Shuyuan Xian, Yifan Liu, Minghao Jin, Xinkun Zhang, Shaofeng Chen, Xi Yue, Wei Zhang, Jianyu Lu, Huizhen Liu, Zongqiang Huang, Hao Zhang, Huabin Yin

**Affiliations:** ^1^ Department of Orthopedics, School of Medicine, Shanghai General Hospital, Shanghai Jiaotong University, Shanghai, China; ^2^ Department of Orthopedics, The First Affiliated Hospital of Zhengzhou University, Zhengzhou, China; ^3^ Tongji University School of Medicine, Shanghai, China; ^4^ Shanghai Jiao Tong University School of Medicine, Shanghai, China; ^5^ Department of Orthopedics, The First Affiliated Hospital of Naval Medical University, Shanghai, China; ^6^ Department of Burn Surgery, The First Affiliated Hospital of Naval Medical University, Shanghai, China; ^7^ Department of Orthopedics, Naval Medical Center of PLA, Second Military Medical University, Shanghai, China

**Keywords:** Ewing sarcoma, EWS/FLI, PHLDA1, CC chemokine receptors, infiltrating T-lymphocytes

## Abstract

**Background:** The molecular mechanisms of EWS-FLI-mediating target genes and downstream pathways may provide a new way in the targeted therapy of Ewing sarcoma. Meanwhile, enhancers transcript non-coding RNAs, known as enhancer RNAs (eRNAs), which may serve as potential diagnosis markers and therapeutic targets in Ewing sarcoma.

**Materials and methods:** Differentially expressed genes (DEGs) were identified between 85 Ewing sarcoma samples downloaded from the Treehouse database and 3 normal bone samples downloaded from the Sequence Read Archive database. Included in DEGs, differentially expressed eRNAs (DEeRNAs) and target genes corresponding to DEeRNAs (DETGs), as well as the differentially expressed TFs, were annotated. Then, cell type identification by estimating relative subsets of known RNA transcripts (CIBERSORT) was used to infer portions of infiltrating immune cells in Ewing sarcoma and normal bone samples. To evaluate the prognostic value of DEeRNAs and immune function, cross validation, independent prognosis analysis, and Kaplan–Meier survival analysis were implemented using sarcoma samples from the Cancer Genome Atlas database. Next, hallmarks of cancer by gene set variation analysis (GSVA) and immune gene sets by single-sample gene set enrichment analysis (ssGSEA) were identified to be significantly associated with Ewing sarcoma. After screening by co-expression analysis, most significant DEeRNAs, DETGs and DETFs, immune cells, immune gene sets, and hallmarks of cancer were merged to construct a co-expression regulatory network to eventually identify the key DEeRNAs in tumorigenesis of Ewing sarcoma. Moreover, Connectivity Map Analysis was utilized to identify small molecules targeting Ewing sarcoma. External validation based on multidimensional online databases and scRNA-seq analysis were used to verify our key findings.

**Results:** A six-different-dimension regulatory network was constructed based on 17 DEeRNAs, 29 DETFs, 9 DETGs, 5 immune cells, 24 immune gene sets, and 8 hallmarks of cancer. Four key DEeRNAs (CCR1, CD3D, PHLDA1, and RASD1) showed significant co-expression relationships in the network. Connectivity Map Analysis screened two candidate compounds, MS-275 and pyrvinium, that might target Ewing sarcoma. PHLDA1 (key DEeRNA) was extensively expressed in cancer stem cells of Ewing sarcoma, which might play a critical role in the tumorigenesis of Ewing sarcoma.

**Conclusion:** PHLDA1 is a key regulator in the tumorigenesis and progression of Ewing sarcoma. PHLDA1 is directly repressed by EWS/FLI1 protein and low expression of FOSL2, resulting in the deregulation of FOX proteins and CC chemokine receptors. The decrease of infiltrating T‐lymphocytes and TNFA signaling may promote tumorigenesis and progression of Ewing sarcoma.

## Introduction

Ewing sarcoma is an aggressive tumor, which typically affects bones and soft tissue in children, adolescents, and young adults (Grunewald et al., 2018). With significant racial disparity, the overall incidence for Ewing sarcoma is ∼1.5 cases per million in Europe, and the peak age is 15 years old (Jawad et al., 2009). Ewing sarcoma is also the second common bone cancer (Ferguson and Turner, 2018) and it usually develops in the diaphysis of bones and metastasizes to lungs and bones. Besides, the primary tumor site varies with age, older patients (20–24 years of age) with a higher proportion of pelvic and axial primary tumors, metastatic diseases, and worse outcomes (Worch et al., 2018). Treatment of patients with Ewing sarcoma includes surgery, chemotherapy, and/or radiation therapy and so on (Grunewald et al., 2018). Currently, the 5-year overall survival is 65–75 percent for patients with localized disease. However, patients with metastatic disease have a strikingly lower 5-year overall survival of less than 30 percent, and those with isolated pulmonary metastasis have approximately 50 percent 5-year overall survival (Gaspar et al., 2015).

Ewing sarcoma is driven by a recurrent t (11; 22) (q24; q12) chromosomal translocation (Aurias et al., 1984) that results in the FET–ETS fusions. The most common fusion is EWS–FLI1 (Delattre et al., 1992), which encodes an oncogenic transcription factor (May et al., 1993), regulating different target genes (Cidre-Aranaz and Alonso, 2015) governing the initiation and progression of Ewing sarcoma (Riggi et al., 2014). Therefore, the molecular mechanisms of EWS-FLI–mediating target genes and downstream pathways may provide a new way in the targeted therapy of Ewing sarcoma.

Enhancers are discrete DNA regulatory elements with specific sequence motifs; they interact with target gene promoters and then enhance the transcription of target genes (Blackwood and Kadonaga, 1998). Meanwhile, enhancers also transcript non-coding RNAs, known as enhancer RNAs (eRNAs) (Kim et al., 2010). Recent progress have found that the transcription of active enhancer mostly initiates cell transcription and 40,000–65,000 eRNAs express in human cells (Andersson et al., 2014; Arner et al., 2015; Lee et al., 2020). Besides the direct mechanism, eRNA can also be elicited by tissue-specific transcription factors (TFs). Importantly, activation of tumorigenesis often converges to the destabilization of eRNAs (Zhang et al., 2019; Lee et al., 2020). However, the functional mechanisms of eRNAs in Ewing sarcoma are still unknown. We proposed that eRNAs may serve as potential diagnosis markers and therapeutic targets in Ewing sarcoma.

In this study, based on an integrated bioinformatics analysis, differential expressed eRNAs, transcription factors, hallmark signaling pathways, and immune cells/functions were identified between Ewing sarcoma samples and normal bone samples. Moreover, we also constructed a complete regulatory network to reveal the potential upstream and downstream mechanisms of further exploring the prognostic biomarkers and treatment targets, which provided a basis and reference for the prognostic risk of Ewing sarcoma tumorigenesis.

## Materials and methods

### Data collection

RNA-sequencing (RNA-seq) data of 85 Ewing sarcoma samples were downloaded from Treehouse database (https://treehousegenomics.soe.ucsc.edu/public-data/#datasets), an RNA database of children’s tumors. RNA-seq data of 3 normal bone samples were downloaded from SRA database (https://www.ncbi.nlm.nih.gov/sra/). For validation, we also obtained gene expression profiles of 256 sarcoma samples from TCGA database (https://tcga-data.nci.nih.gov). Also single-cell RNA sequencing (scRNA-seq) data of GSE146221 were downloaded from Gene Expression Omnibus (GEO) database (https://www.ncbi.nlm.nih.gov/geo/query/acc.cgi?acc=GSE146221) to verify our results. Batch effects of these RNA-seq data were reduced using normalization and batch-effect correction methods.

Next, the eRNA expression profiles of Ewing sarcoma and the target gene list corresponding to eRNAs were downloaded from eRNA in cancer (eRic) database (https://hanlab.uth.edu/eRic/) (Zhang et al., 2019), which benefits researchers to obtain eRNA expression profile, as well as the target genes and drug response of eRNA across TCGA samples. Besides, based on the gene location in hg38 genome, ChIP seeker package was utilized to annotate the official gene symbol of each eRNA (Yu et al., 2015).

Moreover, expression profiles of 318 transcription factors (TFs) were downloaded from Cistrome database (http://cistrome.org/) (Zheng et al., 2019). 50 hallmarks of cancer and 29 immune gene sets were obtained from the Molecular Signatures Database (MSigDB) (http://software.broadinstitute.org/gsea/msigdb) (Liberzon et al., 2015). This study was approved by the Ethics Committee of Tongji University School of Medicine.

### Differential expression analysis

First off, differential expression analysis was conducted to identify differentially expressed genes (DEGs) between Ewing sarcoma samples and normal bone samples by utilizing the Linear Models for Microarray Data (limma) package (Smyth, 2004). Specifically, DEGs were distinguished according to | Log2 fold-change (FC) | > 1 and false discovery rate (FDR) < 0.05. Also, Gene Ontology (GO) and Kyoto Encyclopedia of Genes and Genomes (KEGG) enrichment analyses were conducted to reveal the biological function of DEGs. Likewise, differentially expressed eRNAs (DEeRNAs) and target genes (DETGs), as well as the differentially expressed TFs between Ewing sarcoma samples and normal bone samples were also identified based on the same criteria.

### Construction of prognostic prediction model in sarcoma

To evaluate the prognostic value of DEeRNAs, we used sarcoma samples from TCGA database to conduct cross-validation and independent prognosis analysis. The sarcoma samples were randomly assigned into training set (156 samples) and testing set (100 samples). Training set was utilized to construct the prognostic prediction model, while testing set was utilized to evaluate the prediction model.

Before constructing the prognostic prediction model, lasso regression was applied to avoid overfitting. Then, univariate Cox regression analysis was performed to select DEeRNAs in relation to prognosis. The DEeRNAs independently associated with prognosis, screened by multivariate Cox regression once again, were eventually integrated into the prognostic prediction model. Thus, the risk score of each sarcoma sample was calculated according to the following formula:

$$Risk\;Score_{i}=\beta_{1}\times gene_{1}+\beta_{2}\times gene_{2}+\beta_{3}\times gene_{3}+...+\beta_{j}\times gene_{j}.$$

Among the formula, “i” was the order number of sarcoma samples, while “j” was the quantity of DEeRNAs in this model. “β” was the regression coefficient of corresponding DEeRNAs. Each sarcoma sample was given a risk score, and based on the mean of risk scores, 256 sarcoma samples was classified as high-risk group and low-risk group. The same was true in training set and testing set. Through ranking risk score of each sarcoma sample, scatter dot plot and heatmap were delineated to display the survival time and the expression of independent prognostic factors in high-risk group and low-risk group. Additionally, receiver operator characteristic (ROC) curve was conducted to evaluate the efficiency of the prediction model. In high-risk group and low-risk group, function enrichment analysis was also conducted using GO and KEGG analysis, as well as the hallmark of cancer gene sets.

### Validation of immune clustering among DEeRNAs

To infer portions of infiltrating immune cells in Ewing sarcoma and normal bone samples, expression of DEeRNAs between Ewing sarcoma samples and normal bone samples as well as the correlation in 8 immune cell types were identified using cell-type identification by estimating the relative subsets of RNA transcripts (CIBERSORT). CIBERSORT was performed with 1,000 permutations, where a threshold <0.05 was recommended. Also, correlation analysis was applied to infer the associations between different types of immune cells.

To validate the prognostic value of immune proportions, CIBERSORT was also implemented in 221 sarcoma samples in TCGA database after removing the missing data. Defined by the primary gene signature file LM22 of CIBERSORT, 22 types of immune cells were identified. Through single-sample gene set enrichment analysis (ssGSEA), the immune infiltration degrees of 29 types of immune cells were detected using 29 immune gene sets from MSigDB. Eventually, Kaplan–Meier survival analysis was utilized to display the correlation between survival and immune proportions in sarcoma samples.

### Identification of differentially expressed hallmarks of cancer and immune gene sets

Gene Set Variation Analysis (GSVA) (Hanzelmann et al., 2013) was conducted to detect the expression of hallmarks of cancer in Ewing sarcoma and normal bone samples. Then, differential expression patterns of 50 hallmarks of cancer between Ewing sarcoma and normal bone samples were determined by differential expression analysis using limma R package (Smyth, 2004). The immune infiltration degrees of 29 types of immune cells in Ewing sarcoma and normal bone samples were detected using ssGSEA based on their specific surface markers (Barbie et al., 2009).

### Construction of DEeRNA regulatory network for Ewing sarcoma oncogenesis

First of all, DEeRNAs and DETGs annotated by eRic database, as well as DETFs were retrieved from the above screening. Then, differentially expressed hallmarks of cancer were quantified as continuous variables by GSVA, and immune cells and gene sets were separately obtained from CIBERSORT and ssGSEA. Subsequently, co-expression analysis was conducted among the aforementioned factors, which were illustrated in different colors. Purple indicated the immune cell types by CIBERSORT, blue indicated the hallmarks of cancer by GSVA, indigo blue indicated the immune gene sets by ssGSEA, yellow indicated potential upstream DETFs of DEeRNAs, and pink indicated potential DETGs of DEeRNAs. The interaction pairs between DEeRNAs and DETFs, DETGs, immune cell types by CIBERSORT, hallmarks of cancer by GSVA, and immune gene sets by ssGSEA were utilized to construct the regulatory network for Ewing sarcoma oncogenesis. In the network, we set thresholds as cor. (correlation coefficient) > 0.85 and *p* < 0.05 between DEeRNAs and DETGs; cor. > 0.70 and *p* < 0.05 between DEeRNAs and DETFs; cor. > 0.50 and *p* < 0.05 between DEeRNAs and infiltrating immune cells; cor. > 0.50 and *p* < 0.05 between DEeRNAs and immune gene sets; cor. > 0.60 and *p* < 0.05 between DEeRNAs and hallmarks of cancer. Besides, the Pearson co-expression analysis was also utilized to estimate the correlation between the six components in the regulatory network.

### Identification of candidate small-molecule drugs

In the Connectivity Map (CMap) database (https://portals.broadinstitute.org/cmap/) (Lamb et al., 2006), DEG maps were utilized to predict the associations between small molecule drugs and various diseases. The positive score was the same as the reference gene expression profile, whereas the negative score may be the opposite. Here, CMap was used to determine small molecule drugs that may target Ewing sarcoma based on the expression profiles. Specifically, the database was utilized to screen enrichment fractions < -0.85 and *p* < 0.05, and small molecule drugs with negative scores were considered as candidate therapeutic molecules.

### ATAC-seq validation of key DEeRNAs

Assay for Transposase Accessible Chromatin with high-throughput sequencing (ATAC-seq) data of key DEeRNAs were obtained from chromatin accessibility landscape of primary human cancers (https://gdc.cancer.gov/about-data/publications/ATACseq-AWG), which were then used to identify the chromatin accessibility in the location of these DEeRNAs (Corces et al., 2018).

### External validation

To further demonstrate the reliability of our findings, multidimensional external validation was conducted based on multiple online databases. First off, the Human Protein Atlas (Uhlen et al., 2015), cBioportal (Cerami et al., 2012), and Oncomine (Rhodes et al., 2004) databases were used to compare the expression of DEeRNAs between normal and pathological tissues. Besides, Encyclopedia of Cancer Cell Lines (CCLE) (Ghandi et al., 2019) was used to show the expression of DEeRNAs across various different cancer cell lines. Also, Gene Expression Profiling Interactive Analysis (GEPIA) was a web-based tool to conduct survival analysis of single gene (Tang et al., 2017; Li et al., 2021).

Moreover, CR Cistrome database (http://cistrome.org/db/#/) (Wang Q et al., 2014)was applied to elucidate the interaction between DETFs and DEeRNAs in the chromatin level, based on chromatin-immunoprecipitation followed by sequencing (ChIP-seq) for Histone 3 Lysine 27 acetylation (H3K27ac). Furthermore, eRic database (Zhang et al., 2019) (https://hanlab.uth.edu/eRic/) was utilized to validate the expression, clinical relevance, target genes, and drug response of DEeRNAs.

### Single-cell RNA sequencing transcriptome analysis

The single-cell RNA sequencing (scRNA-seq) data of GSE146221 were downloaded from Gene Expression Omnibus (GEO) database (https://www.ncbi.nlm.nih.gov/geo/query/acc.cgi?acc=GSE146221), which included Ewing sarcoma cell lines CHLA9, CHLA10, and TC71 (Miller et al., 2020). All data were integrated by “IntegrateData” function and analyzed by the R toolkit Seurat (http://satijalab.org/seurat/). Those cells were extracted for the following analysis which had more than 100,000 transcripts expressing. After the top 2,000 variable genes were filtered *via* “vst” method, “FindConservedMarkers,” and “FindMarkers” function, the marker genes of each cell type were identified. The MKI67, CD44, CD24, and PROM1, markers of tumor stem cells, were also utilized to determine the tumor stem cells. Data dimensionality were reduced by principal component analysis (PCA) and the top 20 principal components (PCs) were extracted for the next clustering analysis and Uniform Manifold Approximation and Projection for Dimension Reduction (UMAP) analysis. “CellCycleScoring” function and markers of phases were utilized to visualize the cell cycle stage. At last, “iTALK” package (Wang et al., 2019) was used to identify the ligand and receptor pairs in different cell types, and the “edgebundleR” package (https://github.com/garthtarr/edgebundleR) was used to visualize the intercellular communication.

### Statistical analysis

All statistical analyses of this study were conducted by R version 3.6.1 and two-tailed *p* < 0.05 was required for statistical significance.

## Results

### Identification of DEGs and functional enrichment analysis

**FIGURE 1:**
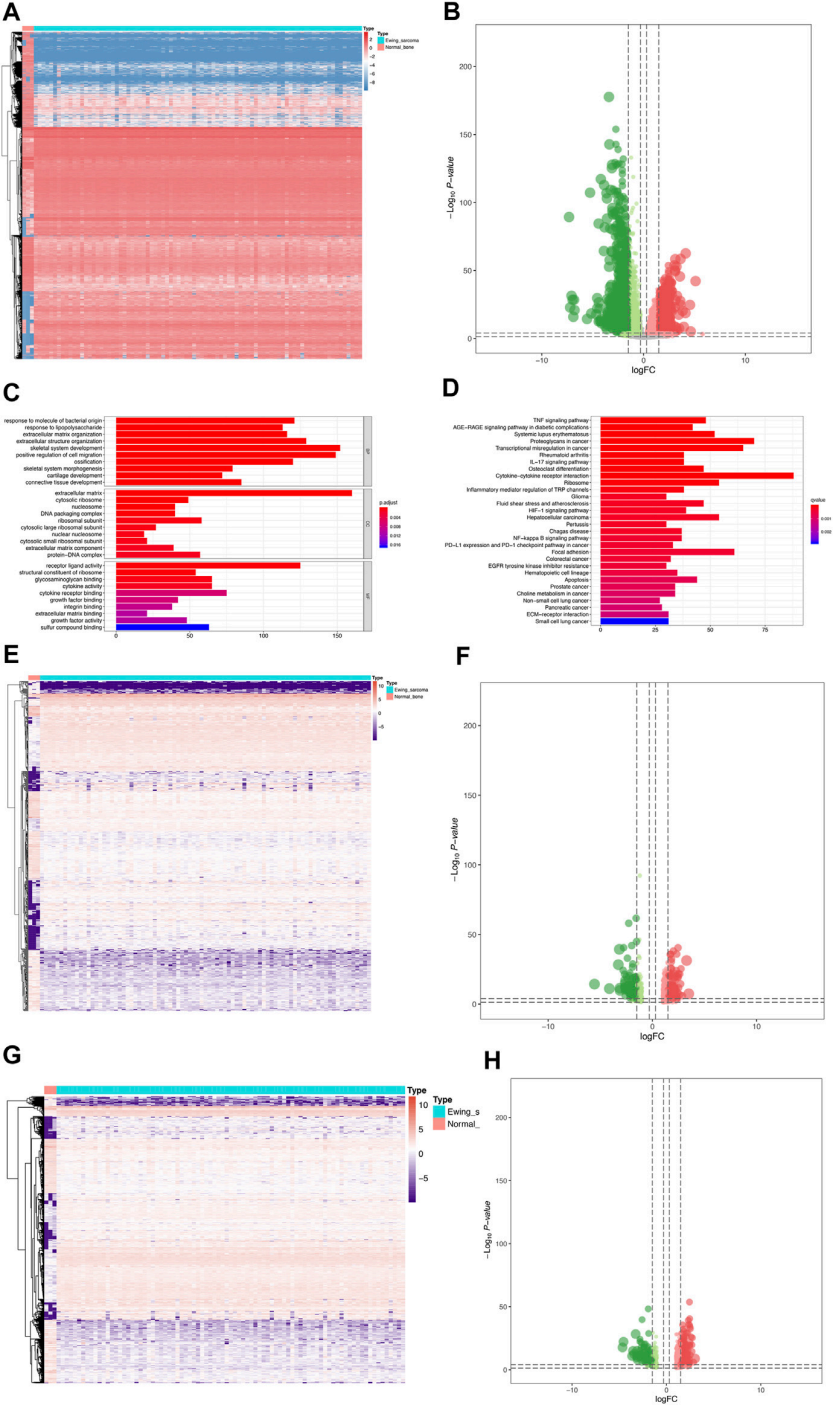
Identification of DEGs, DEeRNAs, and DETFs. **(A)** The DEG analysis between 85 Ewing sarcoma patients and 3 normal samples. **(B)** The volcano plot of a total of 4,941 DEGs identified between Ewing sarcoma samples and normal samples. **(C)** The GO enrichment analysis of DEGs. **(D)** The KEGG enrichment analysis of DEGs. **(E)** The heatmap of 669 DEeRNAs identified between Ewing sarcoma samples and normal bone samples. **(F)** The volcano plot of DEeRNAs. **(G)** The heatmap of 664 DETFs identified between Ewing sarcoma samples and normal bone samples. **(H)**The volcano plot of DETFs.

The analysis process of this study was presented in Supplementary Figure S1. A total of 4,941 DEGs were identified between 85 Ewing sarcoma patients and 3 normal bone samples, the expression of which was illustrated in the heatmap (Figure 1A). The volcano plot of the DEGs was illustrated in Figure 1B. GO and KEGG enrichment analyses were conducted using R’s cluster Profiler software package. The most significant GO items of biological processes (BPs), cellular components (CCs), and molecular functions (MFs) were skeletal system development, extracellular matrix, and positive regulation of cell migration, respectively (Figure 1C). Cytokine–cytokine receptor interaction, proteoglycans in cancer, and transcriptional mis-regulation in cancer were the most critical KEGG pathways, in which most DEGs were enriched (Figure 1D). Furthermore, a total of 669 eRNAs were defined as DEeRNAs between Ewing sarcoma patients and normal bone samples from 5,100 eRNAs, which were illustrated by the heatmap (Figure 1E) and volcano plot (Figure 1F). The heatmap and volcano plot of 664 DETFs identified between Ewing sarcoma patients and normal bone samples were shown in Figures 1G,H.

### Construction of a prognostic prediction model in sarcoma

**FIGURE 2:**
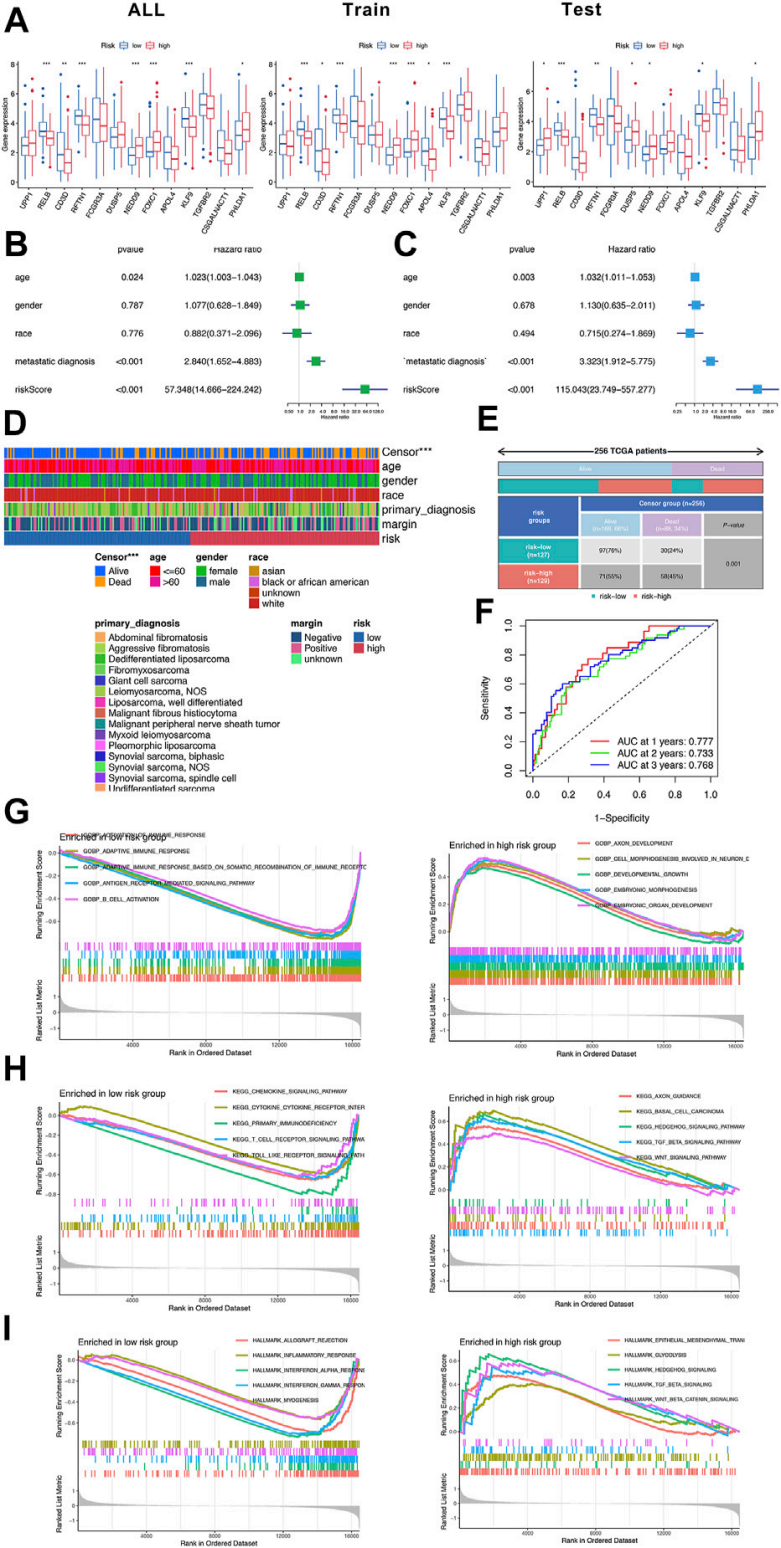
Construction of prognostic prediction model in sarcoma. **(A)** The differential expression of 13 DEeRNAs in the prognostic prediction model. **(B)** The univariate Cox regression analysis of training set. **(C)** The multivariate Cox regression analysis of training set. **(D)** The summary of clinical information of 256 sarcoma samples in TCGA database. **(E)** The classification of sarcoma samples based on the risk score and censor group. **(F)** The ROC curves of the prognostic prediction model at 1-year, 2-years and 3-years **(G)** The GO analysis of low-risk group and high-risk group. **(H)** The KEGG analysis of low-risk group and high-risk group. **(I)** The hallmarks of cancer identified by GSVA in low-risk group and high-risk group.

To evaluate the prognostic value, we used 256 sarcoma samples from TCGA database to conduct cross-validation and independent prognosis analysis. The sarcoma samples were randomly assigned into training set (156 samples) and testing set (100 samples). First off, lasso regression was applied to screen DEeRNAs to avoid overfitting (Supplementary Figures S2A,B). The univariate Cox regression analysis and multivariate Cox regression analysis of training set were displayed in Figures 2B,C. Eventually, 13 DEeRNAs significantly related to prognosis were merged into the prediction model. According to the computing formula of risk score, the sarcoma samples were sorted into high-risk group and low-risk group (Supplementary Figures S2D,E). The differential expression and heatmap of 13 prognostic-related DEeRNAs were illustrated in Figure 2A and Supplementary Figure S2C. Ranking by the risk score of each sample, the scatter dot plot, and the distribution curve were shown in Supplementary Figures S2D,E. The area under the curve (AUC) of ROC curve was 0.725 in all sets, 0.728 both in training set and testing set. Also, in all sets, the AUC of ROC curve was 0.777 at 1-year, 0.733 at 2-years, and 0.768 at 3-years. These values showed a good predictability of the prognostic prediction model in sarcoma.

The function enrichment analysis was also conducted in both high-risk group and low-risk group. In low-risk group, B cell activation and some other adaptive immune response gene sets were enriched in Go analysis; cytokine–cytokine receptor interaction and toll-like receptor signaling pathway were enriched in KEGG analysis, representatively, inflammatory response and myogenesis were enriched in hallmarks of cancer. In high-risk group, embryonic organ development and embryonic morphogenesis gene sets were detected by Go analysis, hedgehog signaling pathway and TGF beta signaling pathway were detected in KEGG analysis as well as hallmarks of cancer (Figures 2G–I).

### CIBERSORT analysis and co-expression analysis of Ewing sarcoma

**FIGURE 3:**
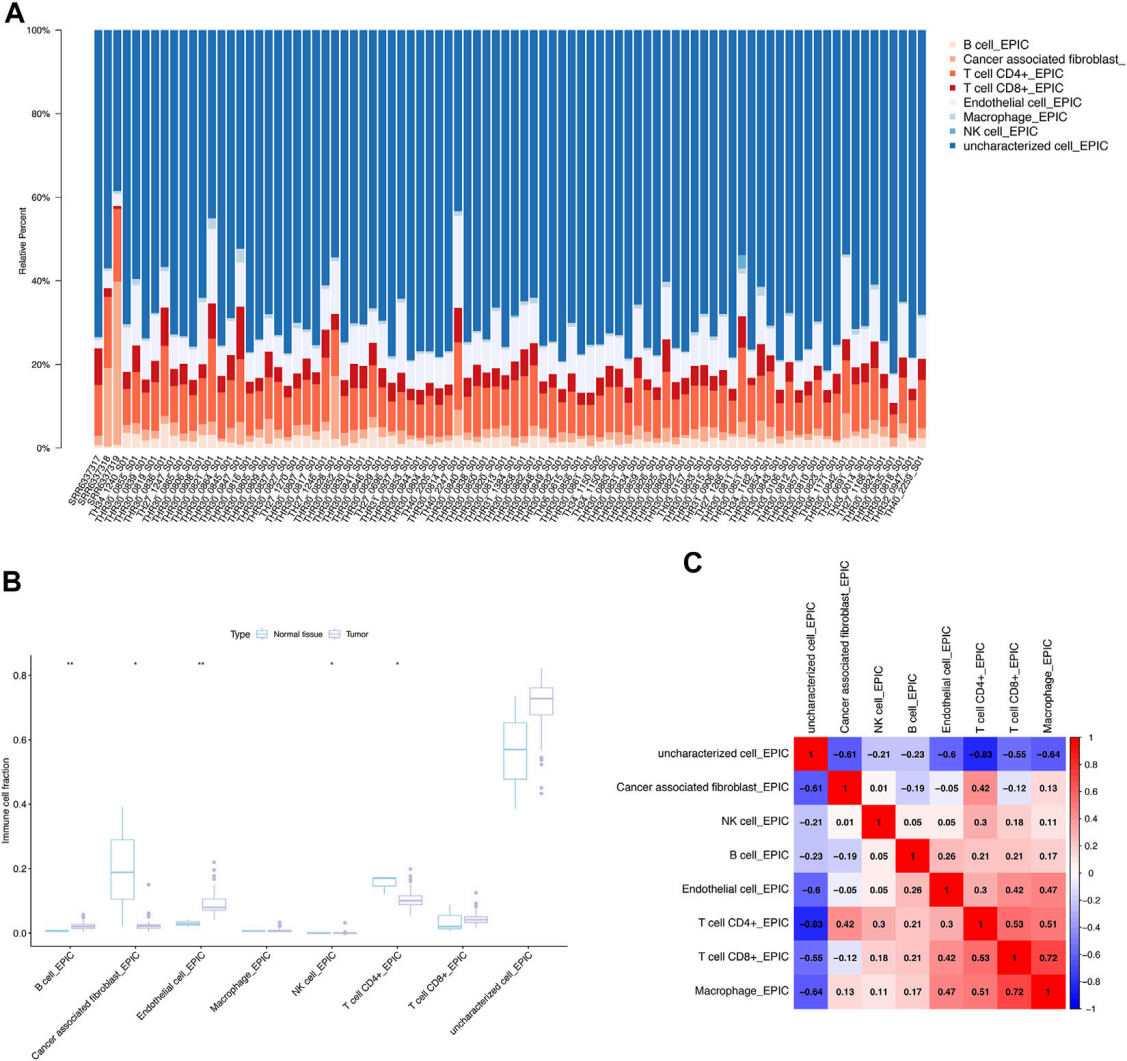
CIBERSORT analysis and co-expression analysis. **(A)** The proportions of 8 immune cells in 85 Ewing sarcoma patients and 3 normal samples explored by CIBERSORT analysis. **(B)** The immune infiltration of Ewing sarcoma and normal bone tissues. **(C)** The co-expression patterns of immune cells in Ewing sarcoma.

We explored the relationship between DEeRNA expression and cancer-infiltrating immune cells, and depicted a summary of the cell compositions in Ewing sarcoma samples and normal bone samples by CIBERSORT algorithm. The proportions of 8 immune cells in 85 Ewing sarcoma patients and 3 normal samples were presented by the bar plot, encompassing B cells, cancer-associated fibroblasts, CD4^+^ T cells, CD8^+^ T cells, endothelial cells, macrophages, NK cells, and uncharacterized cells (Figure 3A). Compared to normal bone tissues, infiltration of endothelial cells (*p* < 0.01) and B cells (*p* < 0.01) was increased, whereas infiltration of cancer-associated fibroblasts (*p* < 0.05), NK cells (*p* < 0.05), and CD4^+^ T cells (*p* < 0.05) was decreased in Ewing sarcoma samples, which suggested that these immune cells had a significantly prognostic value for Ewing sarcoma (Figure 3B). While, the heatmap showed the co-expression patterns between CD4^+^ T cells and CD8^+^ T cells (R = 0.53); CD4^+^ T cells and macrophages (R = 0.51); endothelial cells and macrophages (R = 0.47); CD8^+^ T cells and macrophages (R = 0.72), indicating a strong correlation between these immune cells in Ewing sarcoma (Figure 3C).

### Validation of immune clustering in sarcoma

**FIGURE 4:**
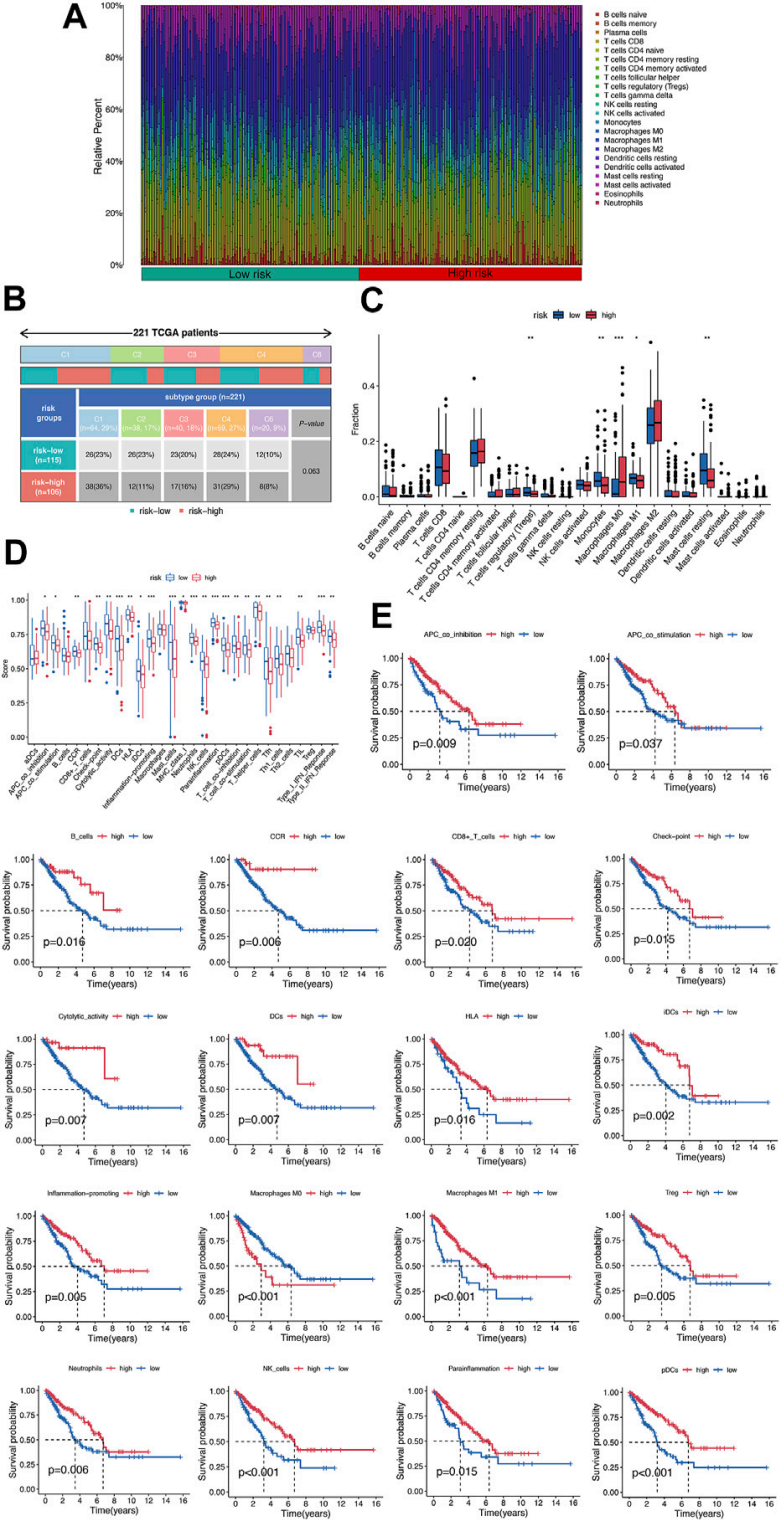
Validation of immune clustering in sarcoma. **(A)** The 22 immune fractions of sarcoma samples identified by CIBERSORT. **(B)** The classification of 221 sarcoma samples based on the risk score and immune subtypes. **(C)** The 22 immune fractions of sarcoma samples classified by risk score. **(D)** The function score of 29 immune gene sets identified by ssGSEA. **(E)** The Kaplan–Meier survival curves of immune gene sets, representatively.

To validate the prognostic value of immune proportions, CIBERSORT was also implemented in 221 sarcoma samples in TCGA database after removing the missing data. The 22 immune fractions of sarcoma samples were displayed in Figure 4A, classified by risk score. The immune subtypes of high-risk group and low risk group sarcoma samples were displayed in Figure 4B. Specifically, T cells CD8, T cells CD4 memory resting, and macrophages M2 comprised a large proportion of immune cells (Figure 4C). On the other hand, 29 immune gene sets were quantified by ssGSEA, which was displayed in Figure 4D. Compared to low-risk group, high-risk group had lower immune function, significant in CCR, check-point, cytolytic activity, DCs, HLA, inflammation promoting, mast cells, neutrophils, NK cells, parainflammation, pDCs, T cells, TIL, Treg, and IFN response. The Kaplan–Meier survival curves below also typically displayed good correlation between immune function and survival in sarcoma samples (Figure 4E).

### Identification of DETFs, differential hallmarks of cancer, and immune gene sets

**FIGURE 5:**
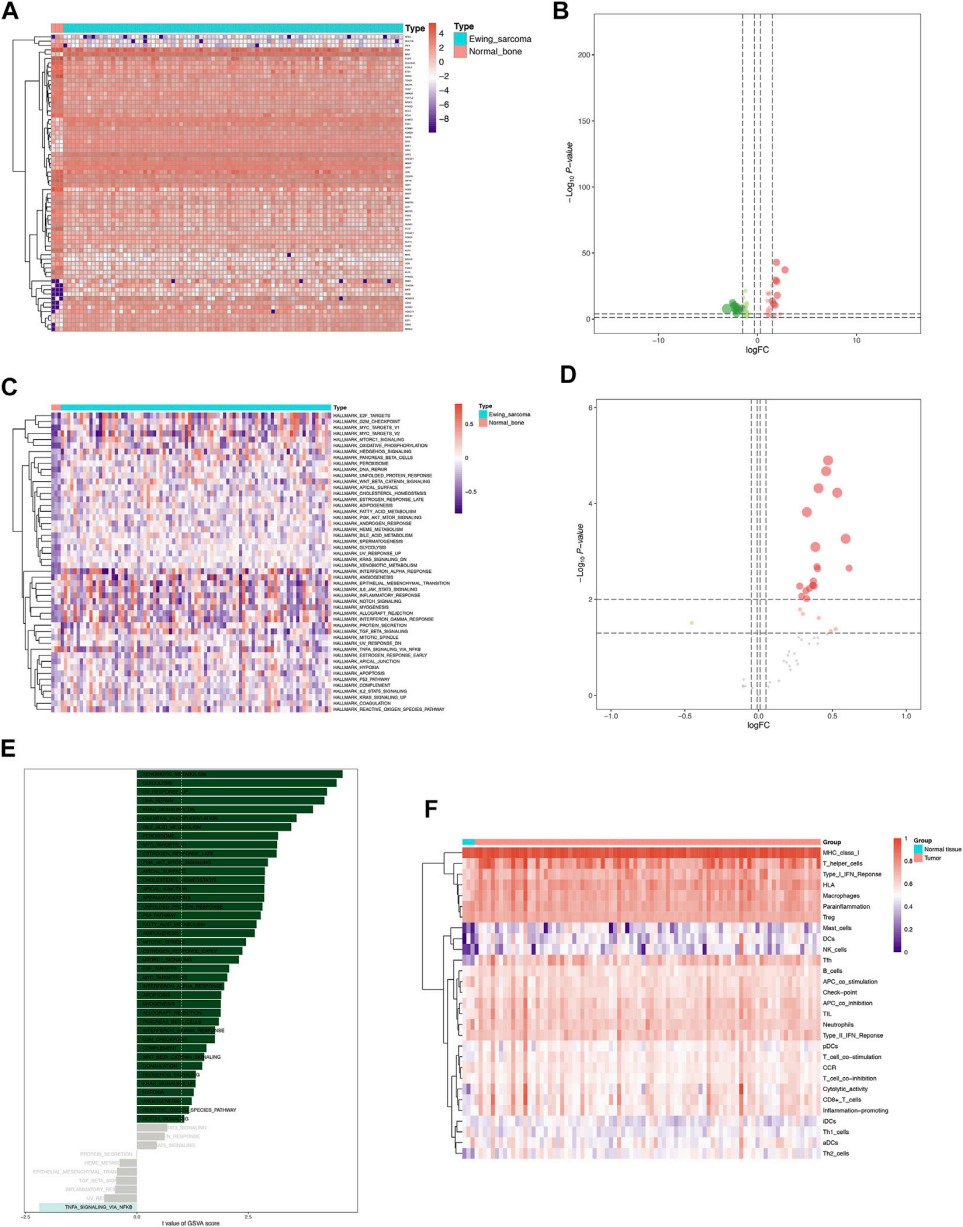
Identification of DETFs, differentially expressed hallmarks of cancer, and immune gene sets co-expressed with DEeRNAs. **(A)** The heatmap of 68 DETFs in Ewing sarcoma samples and normal bone samples. **(B)** The volcano plot of DETFs. **(C)** The heatmap of 21 differentially expressed hallmarks of cancer identified between Ewing sarcoma samples and normal bone samples. **(D)** The volcano plot of differentially expressed hallmarks of cancer. **(E)** The correlation of GSVA score of hallmark pathways and Ewing sarcoma. **(F)** The heatmap of 29 immune-related terms evaluated by ssGSEA between the Ewing sarcoma samples and normal bone samples.

Representatively, the heatmap and volcano plot of 68 DETFs in Ewing sarcoma samples and normal bone samples were shown in Figures 5A,B. A total of 21 differential hallmarks of cancer were identified from 50 hallmark pathways between Ewing sarcoma samples and normal bone samples, which were shown in the heatmap and volcano plot (Figures 5C,D). Besides, the correlation of GSVA score of hallmark pathways and Ewing sarcoma was investigated (Figure 5E). Immune cell infiltration status was evaluated using ssGSEA to validate the associations between the Ewing sarcoma samples and normal bone samples with tumor immune characteristics. Specifically, 29 immune-related terms, or immune functions, were quantified in the heatmap to unravel the abundance of diverse immune cell types in Ewing sarcoma samples and normal bone samples (Figure 5F).

### The network construction and Connectivity Map Analysis

**FIGURE 6:**
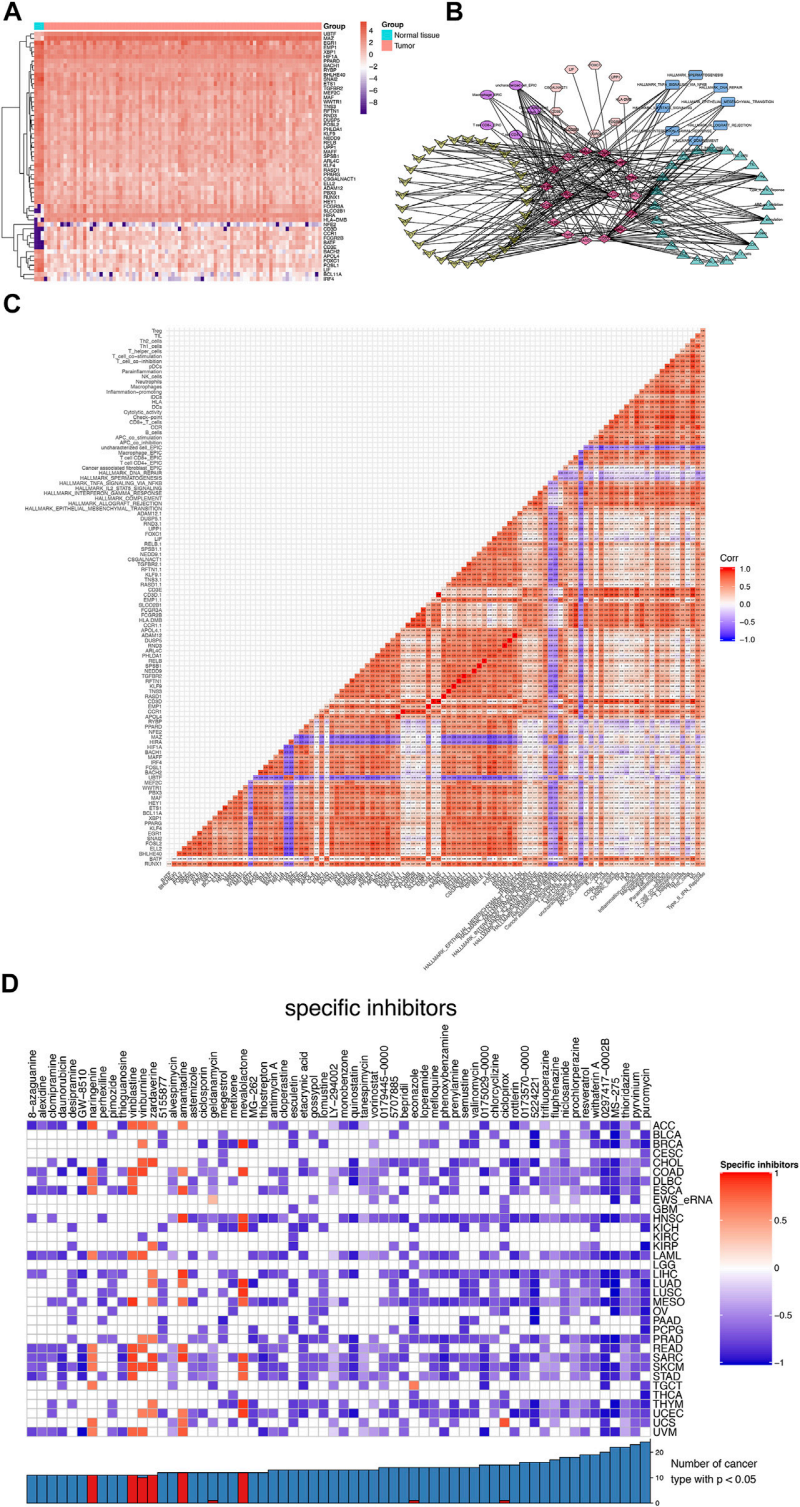
The network construction and Connectivity Map Analysis. **(A)** The heatmap of DEeRNAs, DETFs, and DETGs. **(B)** The six different dimension regulatory network, encompassing 17 DEeRNAs, 29 DETFs, 9 DETGs, 5 immune cells by CIBERSORT, 24 immune gene sets by ssGSEA, and 8 hallmarks of cancer by GSVA. **(C)** The cor-expression heatmap of these components above. **(D)** The CMap analysis of Ewing sarcoma as well as other 33 cancer types.

After the co-expressed analysis, the heatmap showed the expression of most significant DEeRNAs, DETFs, and DETGs in Figure 6A. A total of six different dimension regulatory network was constructed with 17 DEeRNAs, 29 DETFs, 9 DETGs, 5 immune cells by CIBERSORT, 24 immune gene sets by ssGSEA, and 8 hallmarks of cancer by GSVA, which showed the potential regulatory relationships across these factors (Figure 6B). Four key DEeRNAs (CCR1, CD3D, PHLDA1, and RASD1) showed significant co-expression relationships in the six different dimension regulatory network. We supposed that these DEeRNAs may play crucial roles in the tumorigenesis of Ewing sarcoma. Furthermore, the interaction coefficients among these components were illustrated by the heatmap by Pearson correlation analysis (Figure 6C).

The heatmap depicted the enrichment score of each compound analyzed by CMap in Ewing sarcoma, as well in other 33 cancer types (Malta et al., 2018). Importantly, MS-275 and pyrvinium with the highest specificity and the lowest *p* value were considered as the best compounds that might target Ewing sarcoma (Figure 6D).

### ATAC-seq and external validation

**FIGURE 7:**
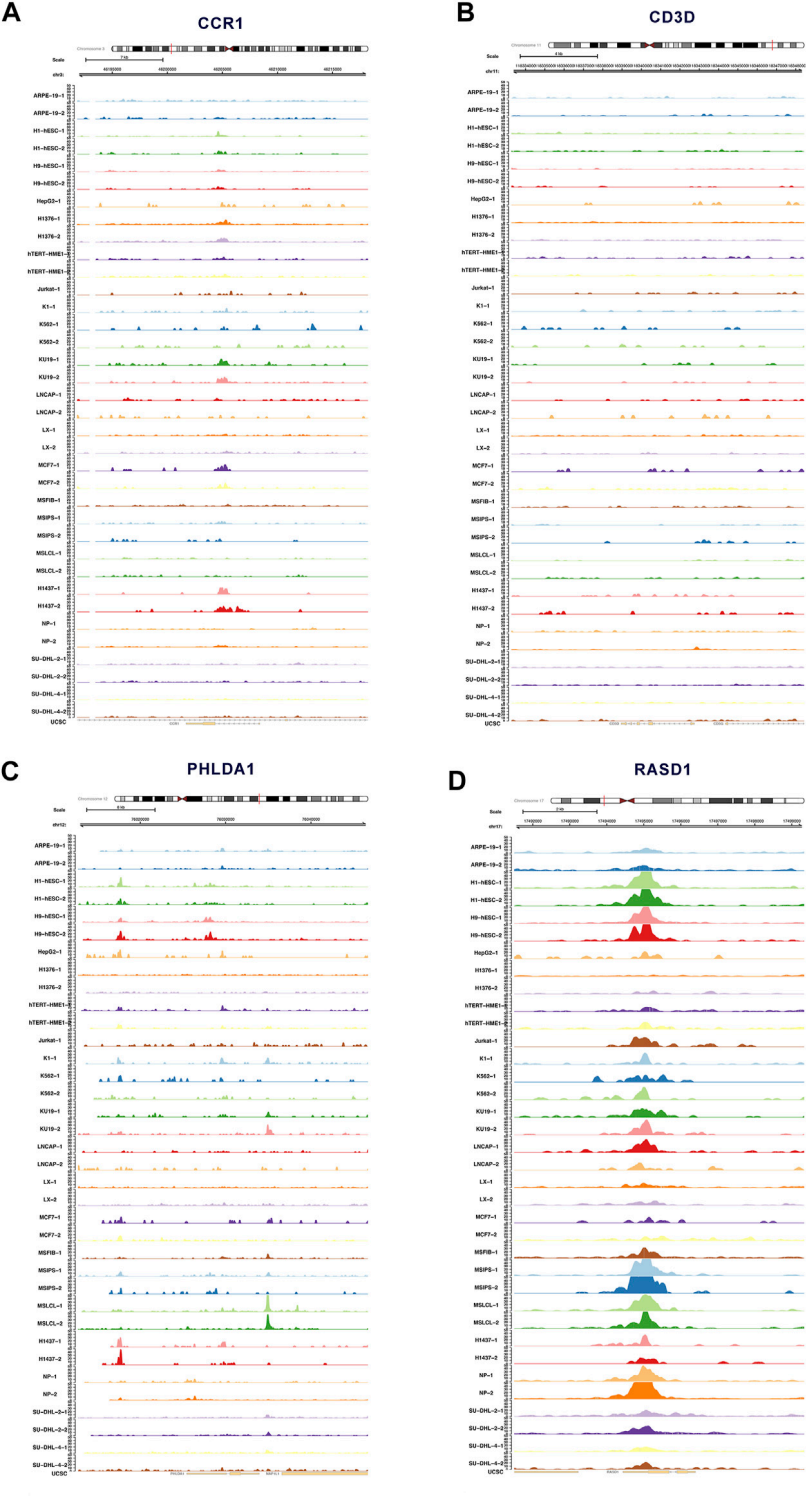
ATAC-seq analysis of key DEeRNAs. **(A)** The accessible chromatin sites of CCR1 analyzed by ATAC-seq. **(B)** The accessible chromatin sites of CD3D analyzed by ATAC-seq. **(C)** The accessible chromatin sites of PHLDA1 analyzed by ATAC-seq. **(D)**The accessible chromatin sites of RASD1 analyzed by ATAC-seq.

Figure 7 depicted the accessible chromatin sites at the key DEeRNAs, including CCR1, CD3D, PHLDA1, and RASD1 (Figures 7A–D). Furthermore, we analyzed data from public databases to assess the prognostic effects of key DEeRNAs and potential regulatory mechanisms in Ewing sarcoma. Based on the human protein atlas database, we examined the expression level of CD3D, MAZ, and PHLDA1 by immunohistochemical (IHC) staining assay and observed that there was medium expression of CD3D, MAZ, and PHLDA1 in normal tissue. Representative IHC images were presented in Supplementary Figure S3. Based on Oncomine database, we identified that expression of CCR1, CD3D, MAZ, PHLDA1, and RASD1 was higher in tumor than that in normal tissue at the pan-cancer level (Supplementary Figures S4A–E). Additionally, expression of CCR1, CD3D, MAZ, PHLDA1, and RASD1 in various different tissues was determined based on CCLE database (Supplementary Figures S5A–E). Taken together, the expression of CCR1, CD3D, PHLDA1, and RASD1 in multiple databases were summarized in Supplementary Table S1. In cBioPortal database, the correlation of mutation count and overall survival of key DEeRNAs were shown in Supplementary Figures S6A–E. Also, the survival analysis of PHLDA1 in GEPIA database was displayed in Supplementary Figure S6G.

To explore the role of enhancer-specific histone in modifications of eRNA transcription, ChIP-seq data of H3K27ac were downloaded and analyzed. The UCSC Genome Browser tracks showed enrichment of H3K27ac on multiple loci in the DEeRNAs (CCR1, CD3D, PHLDA1, and RASD1) (Supplementary Figures S7–S10). Results of external validation in eRic database showed the specific chromatin localization and target genes of the four key DEeRNAs (CCR1, CD3D, PHLDA1, and RASD1), as well as potential drugs that may target these DEeRNAs in different cancers (Supplementary Table S2). We further investigated the expression of key DEeRNAs between tumor and normal samples among different cancer types and identified that the expression level of CCR1, CD3D, PHLDA1, and RASD1 was significantly up-regulated in tumor tissue, as compared with normal tissue. Additionally, prognostic effect of key DEeRNAs was displayed between high expression group and low expression group (Supplementary Figures S11–S14).

### Single-cell RNA-seq transcriptome analysis

**FIGURE 8:**
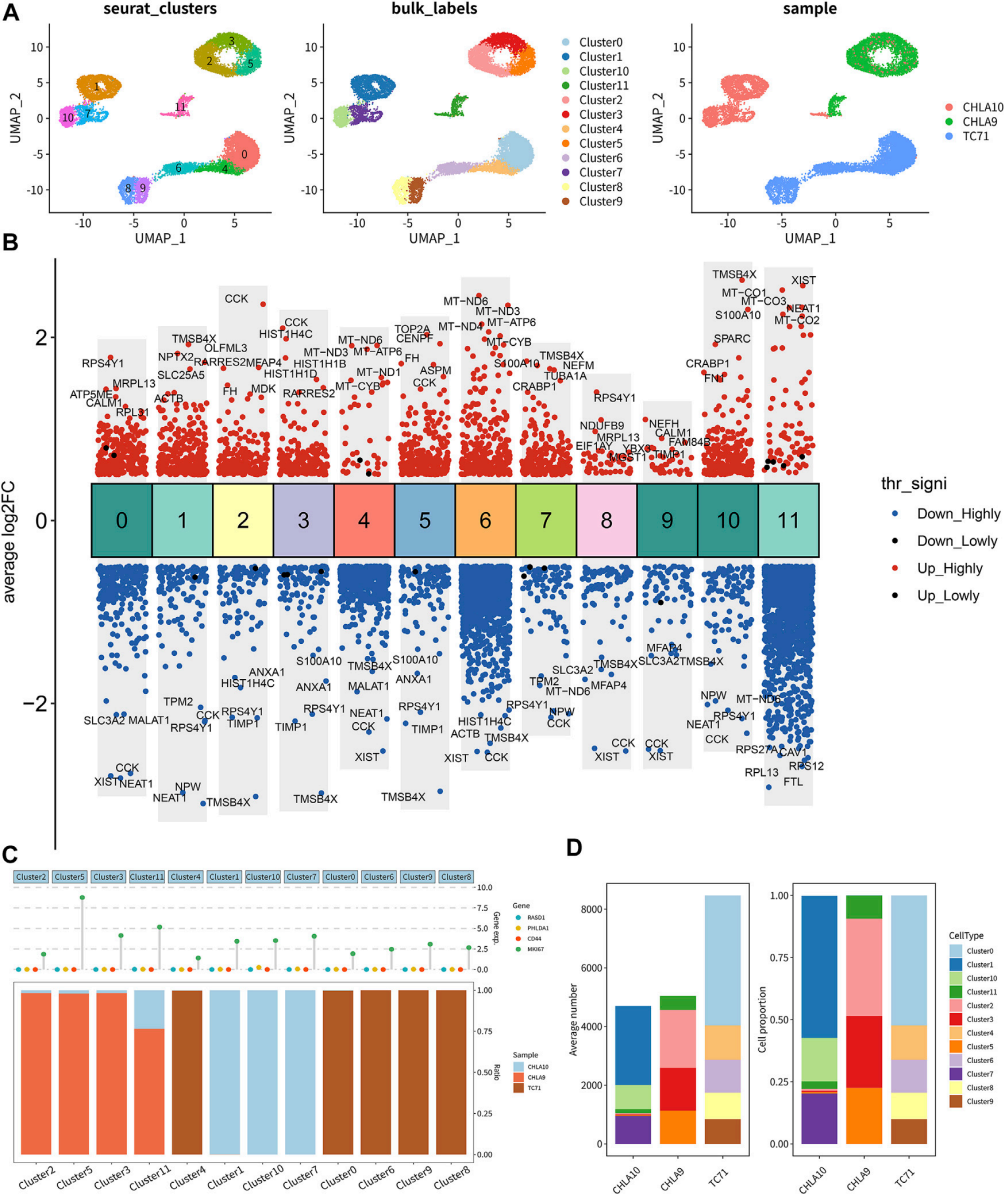
Single-cell transcriptomic analysis of Ewing sarcoma cell lines. **(A)** The distribution of 12 clusters in 3 Ewing sarcoma cell lines (CHLA9, CHLA10, and TC71). **(B)** Gene co-expression of top 5 genes in clusters. **(C)** Significantly up- or down-regulated genes in clusters. **(D)** Cell number and cell proportion of 12 clusters in 3 cell lines.

**FIGURE 9:**
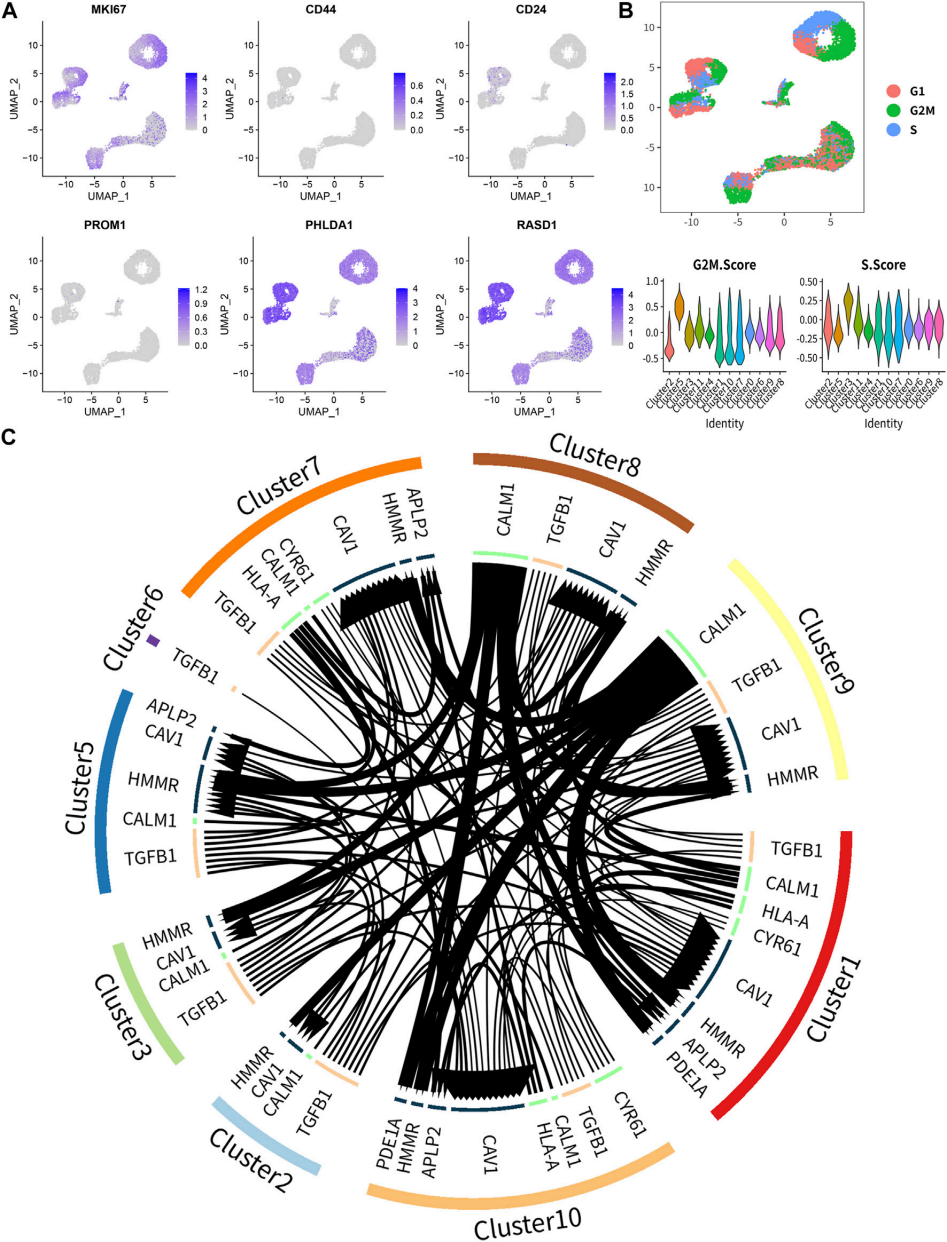
The key biomarkers extensively expressed in Ewing sarcoma stem cell. **(A)** The distribution of marker genes, and MKI67, PHLDA1 and RASD1 were extensively high expressed in all clusters. **(B)** The cell cycle distribution and the cell cycle score in 12 clusters. **(C)** The ligand-receptor pairs among 12 clusters.

Unsupervised clustering clearly identified 12 cell clusters (Figure 8A, left). By utilizing the expression of differentially expressed marker genes, we attributed these clusters to 3 Ewing sarcoma cell lines (CHLA9, CHLA10, and TC71) based on hierarchical similarities (Figure 8A). The heatmap displayed the up- or down-regulated genes in the 12 clusters (Figure 8B). The dot plots showed the proportion of cells expressing tumor stemness-related gene markers (CD44 and MKI67) and key DEeRNAs (PHLDA1 and RASD1) and their scaled relative expression level in 12 cell clusters (Figure 8C). Specifically, MKI67 (a known nuclear marker of proliferation) was highly expressed in all cell clusters, indicating high cellular proliferative activities in these cancer cells. The cell number and proportions of 3 main cell subtypes were quite diverse among the 12 cell clusters (Figure 8D). As demonstrated in the top DEGs, specifically cancer stem lineage clusters expressed high levels of stemness feature genes (MKI67, CD44, CD24, and PROM1) and key DEeRNAs (PHLDA1 and RASD1) (Figure 9A). The cell cycle distribution of 12 cell clusters was shown in the UMAP plot (Figure 9B). Cells within cluster 5 were mainly in G2 phase while cells in cluster 3 were mainly in S phase. The ligand-receptor plot displayed ligand-receptor pairs among those clusters (Figure 9C). All these results showed that PHLDA1 and RASD1 (key DEeRNAs) were extensively expressed in cancer stem cells of Ewing sarcoma, which were potential targets for tumor treatment.

## Discussion

Ewing sarcoma is the second common bone cancer, with strikingly low 5-year overall survival after metastasis (Gaspar et al., 2015). Ewing sarcoma is characteristic with a recurrent chromosomal translocation and the EWS-FLI fusion may provide a new way in the targeted therapy of Ewing sarcoma (Cidre-Aranaz and Alonso, 2015). eRNAs are generated during the transcription of active enhancer (Zhang et al., 2019). In human cancers, eRNAs are specific to tumor types (Lee et al., 2020). Various eRNAs have been demonstrated to be differentially expressed in prostate cancer (Zhao et al., 2016). In breast cancer cells, estrogen-induced transcription of eRNAs was identified to be significantly upregulated (Crudele et al., 2020). Conversely, a recent study showed that expression of eRNAs was significantly decreased in throat cancer (Zhang et al., 2019). Collectively, activation of oncogenes or oncogenic pathways was associated with aberrant generation of eRNAs in human cancers, and eRNAs may play a broad role in the pathophysiology of Ewing sarcoma.

To the best of our knowledge, this is the first study to show DEeRNAs which are potentially engaged in the cellular transition from the normal cells into malignant cells and their potential regulatory relationships in Ewing sarcoma. Herein, an integrated bioinformatics analysis was performed to determine differential eRNA and target gene expression between Ewing sarcoma and normal samples. Differentially infiltrating immune cells were detected by CIBERSORT between Ewing sarcoma samples and normal bone samples. To verify the prognostic power of DEeRNAs and immune proportions, we used sarcoma samples from TCGA database into cross-validation and independent prognosis analysis, as well as Kaplan–Meier survival analysis. In addition, we constructed a DEeRNA co-expressed regulatory network of Ewing sarcoma, encompassing 17 DEeRNAs, 29 DETFs, 9 DETGs, 5 immune cells by CIBERSORT, 24 immune gene sets by ssGSEA, and 8 hallmarks of cancer by GSVA. Importantly, four DEeRNAs (CCR1, CD3D, PHLDA1, and RASD1) were considered to have significant co-expression relationships in the six different dimension regulatory networks. Moreover, Connectivity Map Analysis was applied to pursue small molecules targeting Ewing sarcoma. ATAC-seq data were utilized to provide information on chromatin accessibility of key DEeRNAs. In the end, external validation based on multidimensional online databases and scRNA-seq analysis were used to verify our key findings, which showed that the screened DEeRNAs play a critical role in the tumorigenesis of Ewing sarcoma and could be utilized as important reference markers for future research.

The signal axes of four key eRNAs (CCR1, CD3D, PHLDA1, and RASD1) were as follows: BATF-CCR1-complemen; BATF-CD3D-allograft rejection; FOSL2-RASD1-tnfa signaling *via* NFKB; and FOSL2-PHLDA1-FOXC1-tnfa signaling *via* NFKB. Importantly, signal axis FOSL2-PHLDA1-FOXC1-TNFA signaling *via* NFkB was extracted for the subsequent analyses by theoretical basis and literature review, which will be explained in detail in the following sections as potential mechanism related to the tumorigenesis of Ewing sarcoma. The correlation coefficient between FOSL2 and PHLDA1 was 0.89 (*p* < 0.001); between PHLDA1 and FOXC1 was 0.87 (*p* < 0.001); between PHLDA1 and TNFA signaling *via* NFkB was 0.70605164 (*p* < 0.001). In the interaction and correlation network, PHLDA1 was also related to cancer-associated fibroblasts (R = 0.67; *p* < 0.001) and CCR (R = 0.53; *p* < 0.001).

In Ewing sarcoma, EWS-FLI fusions encode oncogenic proteins functioning as a transcription factor regulating abnormal transcription (Sanchez et al., 2008). Well, a number of studies have described target genes mediated by EWS/ETS proteins. In particular, PHLDA1 has been reported to be few target genes that are directly repressed by the binding of EWS/FLI1 through meta-analysis and experiments *in vitro* (Boro et al., 2012). PHLDA1 (pleckstrin homology-like domain family, member 1) gene is one of the members of the PHLDA gene family (Frank et al., 1999), which has been reported to suppress tumorigenesis (Chen et al., 2018). To be specific, PHLDA1 may repress tumorigenesis by inducing apoptosis and inhibiting cell growth (Neef et al., 2002; Chen et al., 2018). In melanoma (Neef et al., 2002), breast cancer (Nagai et al., 2007), oral cancer (Coutinho-Camillo et al., 2013), and stomach cancers (Zhao et al., 2015), the reduced expression of PHLDA1 has already been described. Moreover, PHLDA1 is not only a tumor suppressor, but also a new targeted therapy to re-sensitize drug-resistant cancer cells (Fearon et al., 2018).

FOS-like antigen 2 (FOSL2) is a member of activator protein-1 (AP-1) transcription factor family (Tulchinsky, 2000), which is involved in cell proliferation, transformation, and death (Shaulian and Karin, 2002). FOSL2 plays a key role in bone development (Bozec et al., 2013). FOSL2 is expressed in stromal cells of human chondroblastic and osteoblastic osteosarcomas, and the deficiency of FOSL2 induces a differentiation defect in osteoblasts both *in vivo* and *in vitro* experiments (Bozec et al., 2010). In addition, FOSL2 has been reported to exert a specific function of mediating TGF-β pathway in extracellular matrix (ECM) remodeling (Busnadiego et al., 2013) and in non-small cell lung cancer (Wang J et al., 2014). In adult T-cell leukemia, aberrantly expressed FOSL2 has been demonstrated to induce CCR4 expression MDM2 (Nakayama et al., 2008).

The Forkhead box C1 (FOXC1) is a member of the Forkhead box (FOX) family, a group of transcription factors and the Fox family are involved in cellular proliferation, differentiation, and death (Lehmann et al., 2003). As a consequence, the deregulation of FOX proteins is able to promote tumorigenesis and cancer progression (M yatt and Lam, 2007). Recently, FOXC1 is demonstrated to be a critical transcriptional regulator for the development and maintenance of hematopoietic stem and progenitor cells (HSPCs) in bone marrow (Omatsu and Nagasawa, 2015). FOXC1 is preferentially expressed to maintain haematopoietic stem and progenitor cells in the adipoosteogenic progenitor CAR cells of developing adult bone marrow (Omatsu et al., 2014). FOXC1 is also able to inhibit CAR cell differentiation into adipocytes, by upregulating CXCL12 and stem cell factor (SCF) (Omatsu et al., 2014). On the other hand, FOXC1 is responsible for governing quiescence by the nuclear factor of activated T-cells 1 (NFATC1) and BMP signaling in stem cells (Wang et al., 2016). Similarly, in basal-like breast cancer (BLBC), FOXC1 may increase cancer stem cell (CSC) properties by cellular mechanisms (Han et al., 2015).

Tumor necrosis factor alpha (TNF‐α) is a cytokine produced by activated macrophages, T lymphocytes, and natural killer (NK) cells, and exerts a wide function in cellular apoptosis and survival, as well as inflammation and immunity. Also, TNF-α is now used in isolated limb perfusion for treatment of soft tissue sarcoma (STS) and other large tumors (Eggermont et al., 2003). Through the activation of nuclear transcription factors, such as NFkB (nuclear factor kappa B) and AP-1, TNF-α is able to modulate the expression of a majority of different genes (Schutze et al., 1992). However, NFkB plays a critical role in preventing cell death induced by TNF-α (Beg and Baltimore, 1996). Aberrant NF-kB expression has been described in many human cancers and tips apoptosis–proliferation balance toward malignant growth (Lin and Karin, 2003).

CC chemokine receptors include CCR 1-10 and CC chemokines are ligands to CCR1-10. The movement of immune cells is driven by CC chemokine receptors and CC chemokines (Hughes and Nibbs, 2018). In cancer, the expression of CC chemokine receptors promotes metastasis and may provide new targets for cancer immunotherapy (Mollica Poeta et al., 2019). CCR7 mediates lymphocyte migration, and CCR9 is involved in rare metastases to the small intestine in melanoma (Zlotnik et al., 2011). In Ewing sarcoma, the expression of CCR5-ligand, CCL5, is positively related to the number of infiltrating CD8^+^ T-lymphocyte and patients with high numbers of infiltrating T-lymphocytes have an overall survival advantage (Berghuis et al., 2011).

Signal axis, FOSL2-PHLDA1-FOXC1-TNFA signaling *via* NFkB, is first reported to be associated with tumorigenesis and progression of Ewing sarcoma. All that being said, the limitations of bioinformatics study are easy to see and unavoidable. First, the sample size of Ewing sarcoma and normal bone samples in our study was limited. Although our results were validated by sarcoma samples, scRNA-seq analysis and multidimensional online databases, larger sample size and more comprehensive data are needed to get more reliable and more accurate results. Second, the direct regulating mechanism of FOSL2-PHLDA1-FOXC1-TNFA signaling *via* NFkB in Ewing sarcoma is unclear. Laboratory-based experiment and clinical study are tremendously needed to explore the direct-action mechanism of the signal axis in Ewing sarcoma. Our hypothesis may just provide a new way for the treatment of Ewing sarcoma.

## Conclusion

In summary, we presume PHLDA1 is a key regulator in the tumorigenesis and progression of Ewing sarcoma. PHLDA1 is directly repressed by the binding of EWS/FLI1 protein and low expression of FOSL2, resulting in the deregulation of FOX proteins and CC chemokine receptors. T-lymphocytes expressing less CC chemokine receptors may not migrate to the tumor site. Inhibition of infiltrating T-lymphocytes and TNFA signaling may promote tumorigenesis and progression of Ewing sarcoma.

## Data Availability

Publicly available datasets were analyzed in this study. These data can be found at: Treehouse database (https://treehousegenomics.soe.ucsc.edu/public-data/#datasets), SRA database (https://www.ncbi.nlm.nih.gov/sra/), TCGA database (https://tcga-data.nci.nih.gov) and GEO database (https://www.ncbi.nlm.nih.gov/geo/query/acc.cgi?acc=GSE146221).

## Supplementary material

Supplementary Figure S1 The schematic diagram of analytic processing.

Supplementary Figure S2 Cross-validation and model diagnosis of sarcoma. **(A)** Coefficient curves of variables in the prognostic prediction model. **(B)** The correlation between λ and partial likehood deviance. At the variable number of 13, the partial likehood deviance was lowest. **(C)** The heatmap of 13 DEeRNAs in all sets, training set, and testing set. **(D)** The scatter dot plot of all sets, training set, and testing set. **(E)** The distribution curve of risk score in all sets, training set, and testing set. **(F)** The area under the curve (AUC) of ROC curve was 0.725 in all sets, 0.728 both in training set and testing set.

Supplementary Figure S3 Human protein atlas database. The medium expression of CD3D **(A)**, MAZ **(B)**, and PHLDA1 **(C)** in normal tissue by immunohistochemical staining assay.

Supplementary Figure S4 Oncomine database. The expression of CCR1 **(A)**, CD3D **(B)**, MAZ **(C)**, PHLDA1 **(D)**, and RASD1 **(E)** in tumor and normal tissues at the pan-cancer level.

Supplementary Figure S5 CCLE database. The expression of CCR1 **(A)**, CD3D **(B)**, MAZ **(C)**, PHLDA1 **(D)**, and RASD1 **(E)** in various different tissues.

Supplementary Figure S6 cBioPortal database and GEPIA database. The correlation of mutation count of CCR1 (A), CD3D (B), RASD1 **(C)**, PHLDA1 **(D)**, and MAZ **(E)** and overall survival. **(F)** The summary of patients’ clinical information and genetic alteration of DEeRNAs. **(G)** The survival analysis of PHLDA1.

Supplementary Figure S7 The ChIP-seq data of CCR1 analyzed by UCSC Genome Browser.

Supplementary Figure S8 The ChIP-seq data of CD3D analyzed by UCSC Genome Browser.

Supplementary Figure S9 The ChIP-seq data of PHLDA1 analyzed by UCSC Genome Browser.

Supplementary Figure S10 The ChIP-seq data of RASD1 analyzed by UCSC Genome Browser.

Supplementary Figure S11 The expression and prognostic effect of CCR1 between tumor and normal samples among different cancer types on eRic database.

Supplementary Figure S12 The expression and prognostic effect of CD3D between tumor and normal samples among different cancer types on eRic database.

Supplementary Figure S13 The expression and prognostic effect of PHLDA1 between tumor and normal samples among different cancer types on eRic database.

Supplementary Figure S14 The expression and prognostic effect of RASD1 between tumor and normal samples among different cancer types on eRic database.
