# Supplementary material for: Repression of enhancer RNA PHLDA1 promotes tumorigenesis and progression of Ewing sarcoma via decreasing infiltrating T‐lymphocytes: A bioinformatic analysis
Source: Front Genet. 2022 Aug 25;13:952162. doi: 10.3389/fgene.2022.952162 (PMC9453160; doi:10.3389/fgene.2022.952162)
Supplement: Supplementary file 2 [file Table1.DOCX]

**Table S1** Summary of external validation results of DEeRNAs based on online databases.

|  | CCR1 | | CD3D | | MAZ | | PHLDA1 | | RASD1 | | Results |
| --- | --- | --- | --- | --- | --- | --- | --- | --- | --- | --- | --- |
|  | N | C | N | C | N | C | N | C | N | C |  |
| The human protein atlas | NA | NA | M | NA | M | NA | M | NA | NA | NA | CD3D, MAZ and PHLDA1 medium-expressed in normal bone marrow (Figure S1). |
| Oncomine | NA | ↑ | NA | ↑ | NA | ↑ | NA | ↑ | NA | ↑ | CCR1, CD3D, MAZ, PHLDA1 and RASD1 over-expressed in sarcoma (Figure S2). |
| CCLE | NA | ↓ | NA | ↓ | NA | ↑ | NA | ↓ | NA | ↓ | CCR1, CD3D, PHLDA1 and RASD1 low-expressed; MAZ  high-expressed in Ewing sarcoma (Figure S3). |
| cBioPortal | NA | NA | NA | NA | NA | NA | NA | NA | NA | NA | The expression data of CCR1, CD3D, MAZ, PHLDA1 and RASD1 in Ewing sarcoma  not available (Figure S4). |

Note: “N” was defined as normal; “C” was defined as cancer; “↑” was defined as a significantly high-expressed gene; “↓” was defined as a significantly low-expressed gene; “M” was defined as medium-expressed gene; “NA” was defined as “Not available”; “ND” was defined as “Not detached”; “-” was defined as a gene with no significant difference in expression.

Abbreviations: CCLE, Cancer Cell Line Encyclopedia.
