# Supplementary material for: Repression of enhancer RNA PHLDA1 promotes tumorigenesis and progression of Ewing sarcoma via decreasing infiltrating T‐lymphocytes: A bioinformatic analysis
Source: Front Genet. 2022 Aug 25;13:952162. doi: 10.3389/fgene.2022.952162 (PMC9453160; doi:10.3389/fgene.2022.952162)
Supplement: Supplementary file 13 [file Table2.DOCX]

**Table S2** Summary of external validation results of DEeRNAs based on eRic database.

| **DEeRNAs** | **CCR1** | **CD3D** | **RASD1** | **PHLDA1** |
| --- | --- | --- | --- | --- |
| **ensemble_ID** | ENSR00000151821 | ENSR00000266115 | ENSR00000282631 | ENSR00000054111; ENSR00000269164 |
| **ensemble_Location** | 3:46189100-46195100 | 11:118329300-118335300 | 17:17498200-17504200 | 12:75937747-75943747; 12:75938400-75944400 |
| **target gene** | CCR1; CCR3 | CD3D; CD3E | RASD1 | KRR1 |
| **target gene_site** | 3:46208396-46201709; 3:46163604-46266706 | 11:118342744-118338954; 11:118304545-118316175 | 17:17496395-17494437 | 12:75511636-75490861 |
| **cancer type** | BLCA; LUAD; THCA | DLBC; THYM; HNSC | PCPG; LGG; THCA; GBM; PRAD | TGCT; HNSC |
| **drug/dataset** | FK866/GDSC;  LY-2183240/CTRP | Olaparib/GDSC;  VAF-347/CTRP | - | FK866/GDSC; PX-12/CTRP; |
